# Preparation and Cytotoxic Evaluation of PGV-1 Derivative, CCA-1.1, as a New Curcumin Analog with Improved-Physicochemical and Pharmacological Properties

**DOI:** 10.34172/apb.2022.063

**Published:** 2021-07-04

**Authors:** Rohmad Yudi Utomo, Febri Wulandari, Dhania Novitasari, Beni Lestari, Ratna Asmah Susidarti, Riris Istighfari Jenie, Jun-ya Kato, Sardjiman Sardjiman, Edy Meiyanto

**Affiliations:** ^1^Cancer Chemoprevention Research Center, Faculty of Pharmacy, Universitas Gadjah Mada (UGM), Sekip Utara, Yogyakarta 55281, Indonesia.; ^2^Medicinal Chemistry Laboratory, Department of Pharmaceutical Chemistry, Faculty of Pharmacy, UGM, Sekip Utara, Yogyakarta 55281, Indonesia.; ^3^Macromolecular Engineering Laboratory, Department of Pharmaceutical Chemistry, Faculty of Pharmacy UGM, Sekip Utara, Yogyakarta 55281, Indonesia.; ^4^Laboratory of Tumor Cell Biology, Division of Bioligical Science, Graduate School of Science and Technology, Nara Institute of Science and Technology, Nara, 630-0192, Japan.

**Keywords:** Curcumin analog, CCA-1.1, NF-κB, Reactive oxygen species, Cytotoxic

## Abstract

***Purpose:*** This study aimed to challenge the anticancer potency of pentagamavunone-1 (PGV- 1) and obtain a new compound (Chemoprevention-Curcumin Analog 1.1, CCA-1.1) with improved chemical and pharmacological properties.

***Methods:*** CCA-1.1 was prepared by changing the ketone group of PGV-1 into a hydroxyl group with NaBH_4_ as the reducing agent. The product was purified under preparative layer chromatography and confirmed with HPLC to show about 93% purity. It was tested for its solubility, stability, and cytotoxic activities on several cancer cells. The structure of the product was characterized using ^1^HNMR, ^13^C-NMR, FT-IR, and HR-mass spectroscopy.

***Results:*** Molecular docking analysis showed that CCA-1.1 performed similar or better interaction to *NF-κB* pathway-related signaling proteins (HER2, EGFR, IKK, ER-alpha, and ER-beta) and reactive oxygen species (ROS) metabolic enzymes (NQO1, NQO2, GSTP1, AKC1R1, and GLO1) compared with PGV-1, indicating that CCA-1.1 exhibits the same or better anticancer activity than PGV-1. CCA-1.1 also showed better solubility and stability than PGV-1 in aqueous solution at pH 1.0–7.4 under light exposure at room temperature. The cytotoxic activities of CCA-1.1 against several (10) cancer cell lines revealed the same or better potency than PGV-1.

***Conclusion:*** In conclusion, CCA-1.1 performs better chemical and anticancer properties than PGV-1 and shows promise as an anticancer agent with high selectivity.

## Introduction

Pentagamavunone-1 (PGV-1) or 2,5-bis(4-hydroxy-3,5-dimethylbenzylidene)cyclopentanone is a new anticancer candidate with a similar chemical structure to curcumin (1,7-bis(4-hydroxy-3-methoxyphenyl)-1,6-heptadiene-3,5-dione). This compound has a strong cytotoxic effect on several cancer cells, such as breast cancer, colon cancer, and blood cancer cells (leukemia) *in vitro*. This compound’s anticancer mechanism is related to apoptosis triggering, cell cycle arrest in the G2/M phase, and increased intracellular reactive oxygen species (ROS) and senescence.^1–4^ PGV-1 induces cell cycle arrest at the G2/M phase differently from conventional anticancer drugs, such as taxanes and vinca alkaloids, and even from curcumin.^1,5,6^ Taxanes and Vinca alkaloids target microtubule,^7,8^ whereas curcumin targets APC (anaphase promoting complex) protein complex, resulting in abrogation of anaphase dynamics.^9^ Interestingly, PGV-1 acts cell cycle termination in prometaphase.^1^ The superiority of PGV-1 was also revealed *in vivo* experiments in which PGV-1 showed a much better tumor-suppressing effect than that of curcumin and did not exhibit any harmful effects in the tested animals.^1^ Therefore, PGV-1 is suitable to be promoted as a cancer drug.

**Figure 1:**
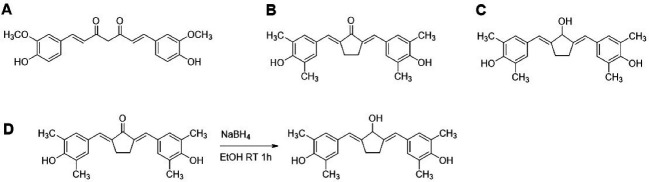
Chemical Structures and CCA-1.1 synthesis scheme. (A) Curcumin or 1,7-bis(4-hydroxy-3-methoxyphenyl)-1,6-heptadiene-3,5-dione, (B) PGV-1 or 2,5-bis(4-hydroxy-3,5-dimethylbenzylidene)cyclopentanone, (C) CCA-1.1 or 2,5-bis(4-hydroxy-3,5-dimethylbenzylidene)cyclopentanol and (D) Synthetic scheme of CCA-1.1.

PGV-1 is relatively easy to be synthesized, but its stability has to be adequately improved. Like curcumin, PGV-1 may undergo decomposition by light or high pH due to the ketone group with the alpha-beta unsaturated (diene) keto system in its structure.^10,11^ Since the alpha-beta unsaturated keto has an essential contribution in its anticancer activity,^12^ it should be maintained. This alpha-beta diene system is susceptible to radical attack because it is connected to the ketone group, a strong electron-withdrawing group.^10^ Therefore, the ketone group tough to be transformed into a hydroxyl group to weaken the electron distribution and produce a new and more stable compound named 2,5-bis(4-hydroxy-3,5-dimethylbenzylidene)cyclopentanol or CCA-1.1 (Chemoprevention-Curcumin Analog 1.1) (Figure 1). Besides, a hydroxyl group in this center position allows this new compound to be reacted with polar compounds, such as polyhydroxy carbon or a peptide. Thus, this new compound can be further derivatized to improve its solubility and bioavailability for therapeutic purposes.

Synthesis of CCA-1.1 from PGV-1 needs a selective reducing agent to reduce ketone into a hydroxyl group (Figure 1). The reduction reaction of PGV-1 to CCA-1.1 is a preferred choice compared to performing condensation reactions such as PGV-1 preparation. The reduction has become one of the reactions typically used in organic chemistry, and several types of reducing agents have been developed for this accomplishment. We are considering the α,β-diene system in the structure of PGV-1, a selective agent that can reduce only the carbonyl ketone group must be used. For this reaction, NaBH_4_ can be chosen instead of H_2_ gas with a palladium catalyst.^13^ NaBH_4_ reagent is a chemo-selective reducing agent and often be used to reduce carbonyl to the hydroxy group, which is carried out under appropriate conditions.^14,15^ The reduction reaction occurs through the addition of hydride (from NaBH_4_) to carbonyl aldehyde or ketone followed by oxygen protonation to yield primary or secondary alcohol.^14^ In this study, we conducted a mild reduction of PGV-1 into CCA-1.1 and evaluated its stability and cytotoxic activity towards several cancer cell lines compared to PGV-1. Before the CCA-1.1 synthesis, we also carried out a molecular docking study of CCA-1.1 compared to PGV-1 against several proteins as the cancer markers, including nuclear factor kappa B (NF-κB)-related signaling proteins and ROS metabolic enzymes.

## Materials and Methods

### Molecular docking analysis

To compare the binding interaction between CCA-1.1 and PGV-1 toward several cancer markers, we performed a molecular docking analysis of several proteins involving ROS metabolic enzymes and NFκB-pathway related proteins based on previously reported works. The computational simulation was performed using licensed-software MOE 2010.10 to simulate molecular binding, calculate root-mean-square deviation (RMSD), and visualize protein-ligand interaction. The ROS metabolic enzymes were represented by NQO1 (PDB ID: 1D4A), NQO2 (PDB ID: 4FGL), AKR1C1 (PDB ID: 1MRQ), GST-P1 (PDB ID: 5J41), and GLO1 (PDB ID: 1QIP) enzymes, and the binding interaction targeted on the cofactor site.^1^ The molecular docking study on NFκB-pathway related proteins were conducted toward HER2 (PDB ID 3PP0), epidermal growth factor receptor (EGFR) (PDB ID: 1XKK), and IKK (PDB ID: 4KIK), focusing on the native ligand binding site.^16^ We also performed molecular docking on ER-alpha (PDB ID: 3ERT) and ER-beta (PDB ID: 5TOA) as the representation of NFκB-pathway related proteins and used site finder mode from MOE due to the unknown reported binding site. The default settings were utilized as long as there is no further explanation. The chemical structure of CCA-1.1 and PGV-1 was created in ChemDraw software and then minimized the structural energy generated for conformational structure in MOE. The docking simulation setting used the MOE default mode, such as triangle matcher and London dG, as the placement setting and scoring method. The force field method was used to refine the docking results from 10 retain settings. The molecular docking results described the affinity represented by the docking score and the binding interaction of each compound with the target proteins.

### Absorption, distribution, metabolism, and excretion (ADME) prediction

Considering that certain compounds’ interaction to the protein targets also depended on their bioavailability profile, we used pkCSM software (http://biosig.unimelb.edu.au/pkcsm/prediction) to compare the ADME profile prediction between PGV-1 and CCA-1.1. CCA-1.1 and PGV-1 have encoded the SMILES code and then generated it for pkCSM analysis. The parameters collected in this study were water solubility, Caco2 permeability, intestinal absorption, volume of distribution at steady state (VDss), and clearance.

### Main materials and general analytical procedures

PGV-1 and curcumin were obtained from the Cancer Chemoprevention Research Center (CCRC), Faculty of Pharmacy, UGM. The reagents and solvents in this experiment were classified as an analytical grade unless otherwise stated. The ^1^H- and ^13^C-NMR spectra of CCA-1.1 were recorded in DMSO-D_6_ with a JNM-ECZ500R (500 MHz, JEOL Ltd., Tokyo, Japan) spectrometer. FT-IR spectrum (KBr disc) was determined using an FT-IR spectrophotometer (Perkin Elmer, USA) at 4000-400 cm^-1^. The ESI-MS spectrum of CCA-1.1 was obtained by using LCMS-2010EV (Shimadzu, Japan). The purity of CCA-1.1 was determined by using a C18 column (Chromosorb) with mobile phase acetonitrile: water (70:30 v/v) in UV-Vis HPLC (Hitachi, Japan).

### Synthesis of CCA-1.1

Synthesis of CCA-1.1 was conducted by reducing the ketone into a hydroxyl group. An amount of 100 mg PGV-1 (0.1 mmol) was dissolved in 10 mL of ethanol. The 11 mg of NaBH_4_ (0.3 mmol) was added then stirred for 1 hour. The crude product was purified using PLC Silica gel 60 F_254_ (Merck) with a mobile phase system of chloroform: methanol (99:1).

### Dissolution and stability test in buffer solution

A stock of curcumin, PGV-1, and CCA-1.1 solution was prepared in DMSO (Merck) at the concentration of 100 mM. The tested solution of curcumin, PGV-1, and CCA-1.1 was prepared by diluting the stock solution in distillate water, phosphate buffer saline pH 1.0, and pH 7.4 up to 100 µM. Each solution in the respected buffer was then evaluated for its stability at ambient temperature and light exposure. It measured the absorbance change at 428, 416, and 404 nm, subsequently over a time-course using a UV-1800 spectrophotometer (Shimadzu, Japan).

### Cell culture, propagation, and cell viability assay

MCF-7/HER2 and MCF-7/EV are HER2 and empty vector-transfected MCF-7 cells obtained from Prof. Yoshio Inouye (Department of Surgery, Toho University School of Medicine, Japan) through Prof. Masashi Kawaichi (Laboratory of Gene Function in Animals, NAIST, Japan). The cells of 4T1, NIH-3T3, and T47D were kindly provided by Prof. Masashi Kawaichi (Laboratory of Gene Function in Animals, NAIST, Japan). K652 cells are the collection from the Laboratory of Tumor Cell Biology, Nara Institute of Science and Technology, Japan. HCC1954 and Caco2 cell lines were given by Dr. dr. Muhammad Hasan Bashari (Faculty of Medicine, Universitas Padjajaran, Indonesia). WiDr cell line was obtained from the Faculty of Medicine, Public Health, and Nursing Universitas Gadjah Mada, Indonesia. Briefly, MCF-7/HER2, MCF-7, NIH-3T3, T47D, MCF-7/EV, and 4T1 cells were cultured in Dulbecco’s Modified Eagles Medium (DMEM) high glucose (Gibco, USA), whereas HCC1954, Caco2, WiDr, K562 cells were grown in Rosewell Park Memorial Institute (RPMI) Medium. Both were supplemented with 10% (v/v) fetal bovine serum (Gibco, USA), HEPES (Sigma, USA), sodium bicarbonate (Sigma, USA), 150 IU/mL penicillin and 150 µg/mL streptomycin (Gibco) and 1.25 µg/mL amphotericin B (Gibco, USA). The cells were maintained at 37 °C with 5% CO_2_ in a humidified atmosphere.

Cells were grown in 24 or 96-well microplate and then cultured for 24 h. Subsequently, cells were treated with PGV-1 or CCA-1.1 at concentration series up to 10 µM, except for NIH-3T3 up to 100 µM and incubated for 24 hours. Untreated cells were used as control. After treatment, viable cells were enumerated using the trypan blue exclusion or quantified under MTT assay.^17^ The viable cells were represented as % cell viability vs. concentration of the sample, and the IC_50_ value or the concentration that inhibits 50% of cell growth was calculated.

### Data analysis

Molecular docking results were validated by determining the RMSD value of conformation bearing the lowest docking score representing the ∆G (kkal/mol). The validity of the molecular docking method was represented as RMSD value < 2. Cytotoxic potencies against several cell lines were statistically analyzed based on the IC_50_ values through linear regression with a *P* value of > 0.05.

## Results

### CCA-1.1 binds to targeted-proteins of PGV-1

**Figure 2:**
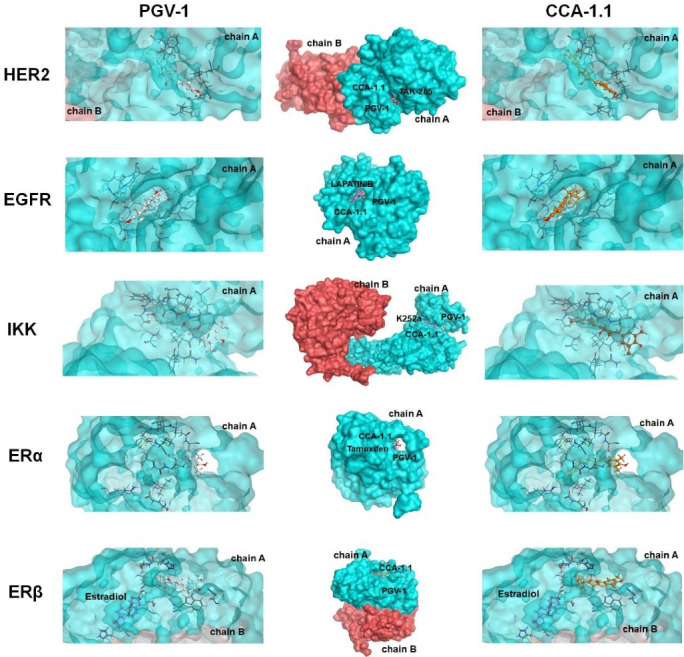
Binding interaction of PGV-1 and CCA-1.1 toward several NFκB pathway-related proteins.

**Figure 3:**
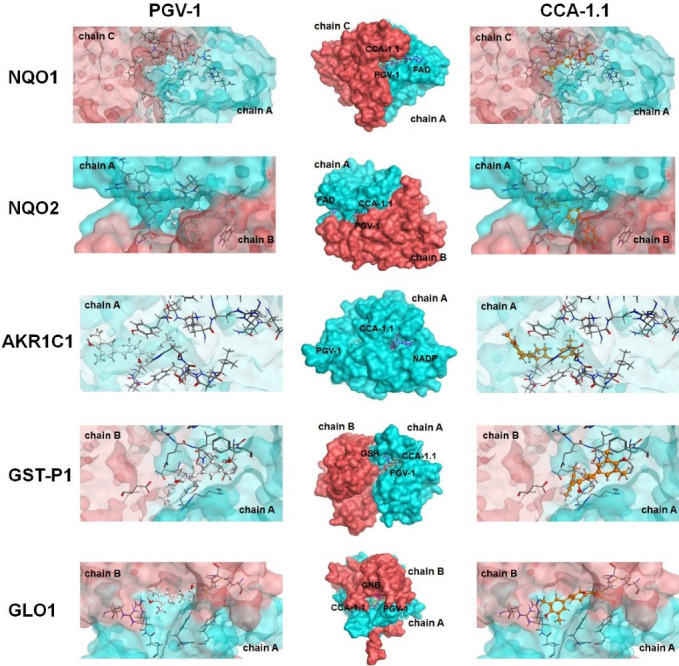
Binding interaction of PGV-1 and CCA-1.1 toward several ROS-metabolic enzymes.

The binding interaction of PGV-1 to several ROS metabolic enzymes and NFκB-pathway related proteins contributed to the potent cytotoxic effect. Molecular docking of several types of target proteins such as HER2, EGFR, IKK, and ER showed that CCA-1.1 exhibited good or better affinity than PGV-1 to all target proteins (Figure 2). Similar to PGV-1, CCA-1.1 also interacted appropriately with ROS metabolic enzymes, such as AKC1R1, GSTP1, GLO1, NQO1, and NQO2 (Figure 3). The molecular docking analysis revealed that the docking score of CCA-1.1 was lower than that of PGV-1 on all target proteins, except IKK, indicating improved binding affinity (Table 1; Table S1 of Supplementary file 1). The hydroxyl group on the cyclic structure formed hydrogen bonds on NQO1, NQO2, GLO1, and ER-alpha, which possibly contributed to the higher affinity of CCA-1.1 than PGV-1. Overall, the molecular docking analysis predicted the higher affinity of CCA-1.1 than PGV-1, making it a more potent cytotoxic agent than PGV-1.

**Table 1:** Docking Score (∆G) (kkal/mol) of PGV-1 and CCA-1.1 towards several proteins

**Protein target**	**Ligand**	
	**PGV-1**	**CCA-1.1**
HER2	-12.69	-13.85
EGFR	-11.69	-12.77
IKK	-11.67	-10.66
ERa	-11.12	-11.58
ERb	-10.97	-11.29
NQO1	-12.54	-14.07
NQO2	-13.60	-14.47
AKR1C1	-13.19	-13.79
GST-P1	-10.57	-11.01
GLO1	-12.79	-14.71

### ADME profile of CCA-1.1 and PGV-1

To predict whether or not the modification of ketone into a hydroxy group improves the bioavailability or not, we expected the ADME profile of PGV-1 and CCA-1.1 by using pkCSM software. The ADME prediction from pkCSM showed that CCA-1.1 exhibited lower log P and higher water solubility than PGV-1. Despite the lower Caco2 permeability and intestinal absorption of CCA-1.1 than PGV-1, the VDss of both compounds were similar **(**Table 2). Summarizing the ADME profile prediction, it can be indicated that CCA-1.1 performed better bioavailability than PGV-1.

**Table 2:** ADME Prediction of PGV-1 and CCA-1.1

**Parameter**	**PGV-1**	**CCA-1.1**
logP	5.161	4.953
Water Solubility	-4.789 log mol/L	-4.624 log mol/L
Caco2 permeability	1.298 log Papp in 10^-6^ cm/s	1.286 log Papp in 10^-6^ cm/s
Intestinal absorption	94.39 % Absorbed	91.87 % Absorbed
Skin Permeability	-3.036 log Kp	-3.092 log Kp
Vdss	0.635 log L/kg	0.658 log L/kg
Total Clearance	0.39 log ml/min/kg	0.891 log ml/min/kg

### Synthesis and structure identification of CCA-1.1

**Figure 4:**
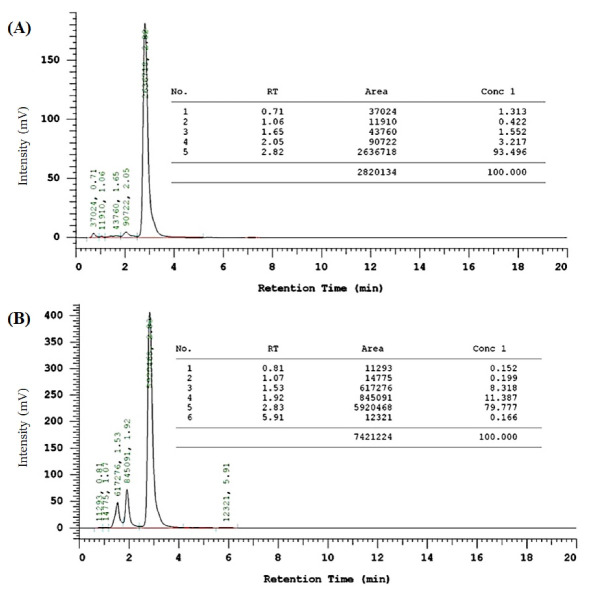
HPLC analysis of CCA-1.1 (A) and PGV-1 (B). Samples in methanol solution were analysed using a mobile phase consisting of acetonitrile:water (70:30 v/v) at flow rate 1 mL·min−1. The retention times for CCA-1.1 and PGV-1 were 2.82 and 2.83 min respectively.

**Figure 5:**
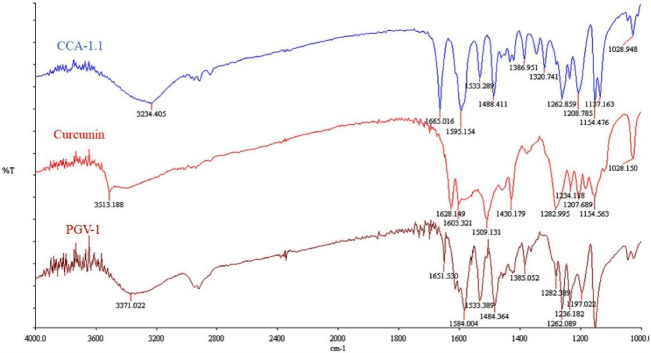
IR Spectra of Curcumin, PGV-1 and CCA-1.1. Samples were prepared in KBr disk and scanned using FT-IR spectrophotometer (Perkin Elmer, USA) at 4000-1000 cm^-1^.

The synthesis of CCA-1.1 was quite simple. The reaction product appears after 20 minutes with the change of color, and we stopped after the changes of color look constant, which occurred in about 1 hour stirring. After PLC purification, we collected the product with a 10% yield by the melting point of 272-277°C. The HPLC analysis of the purified product exhibited the retention time at 2.82 minutes with an abundance of 93.496%, indicating that it showed relatively high purity (Figure 4). The molecular formula of CCA-1.1, which is corresponded to its molecular weight, was established by ESI-MS as C_23_H_26_O_3,_ which showed [M-H]- ion peak at 349.3280 (Figure S1). We confirmed the functional groups of CCA-1.1 through FT-IR analysis, with curcumin and PGV-1 were used as reference (Figure 5). PGV-1 as reference showed several peaks at 3371 cm^-1^ (Ar OH), 2900 cm^-1^ (CH_3_), 1651 cm^-1^ (C = C), 1620 cm^-1^ (R_2_C = O), and 1600-1554 cm^-1^ (Ar C = C). Meanwhile, CCA-1.1 showed peaks at 3100-3500 cm^-1^ (Ar OH dan R_2_C-OH), 2900 cm^-1^ (CH_3_), 1665 cm^-1^ (C = C), and 1595-1560 cm^-1^ (Ar C = C), with no peak at around 1620 cm^-1^ due to the loss of C = O group. In the curcumin spectrum, there was a peak in the most significant C = O group present at 1628 cm^1^. Taken together, the structure of CCA-1.1 was successfully synthesized through PGV-1 reduction, and the result was confirmed basing from on its functional group through IR spectroscopy. The ^1^H-NMR (Figure S2) and ^13^C-NMR (Figure S3) spectra showed five and eight resonance peaks, respectively, confirming that CCA-1.1 is a symmetrical molecule similar to PGV-1 and curcumin. The very downfield broad signal at δ 8.84 in the ^1^H-NMR spectrum was characteristic for aromatic hydroxyl proton. The proton resonance at δ 7.27 ppm (s, 3H) was assigned to aromatic (H-2’ and H-6’) and vinylic (H6) protons. The hydroxymethyl (H-1) and methylene (H-3 and H-4) protons of the cyclopentanol ring appeared at δ 3.30 and 3.02 ppm, respectively. The rest singlet signal at δ 2.20 ppm was referred to two methyl protons attached to the benzene ring at positions 3’ and 5’. In the ^13^C-NMR spectrum, the resonance of C-1, C-2/C-5, and C-3/C-4 of the cyclopentanol ring was represented by the resonance signals δ 124.6, 134.5, and 25.9 ppm, respectively. The chemical shift of C-2/C-5 was high because they are two quaternary sp2 carbon atom of a vinyl moiety. The other vinyl carbon (C-6 and C-7) signal appeared at δ 126.7 ppm. The carbon resonance at δ 132.5, 131.3, and 155.1 ppm were assigned to C-1’, C-2’/C-3’/C-5’/C-6’, and C-4’ of the benzene ring, respectively. The aromatic methyl carbons were observed at δ 14.8 ppm. Basing on the above spectral analysis, we confirmed the synthesis product was 2,5-bis(4-hydroxy-3,5-dimethylbenzylidene)cyclopentanol or CCA-1.1.

### Solubility and stability of CCA-1.1 compared with PGV-1

**Figure 6:**
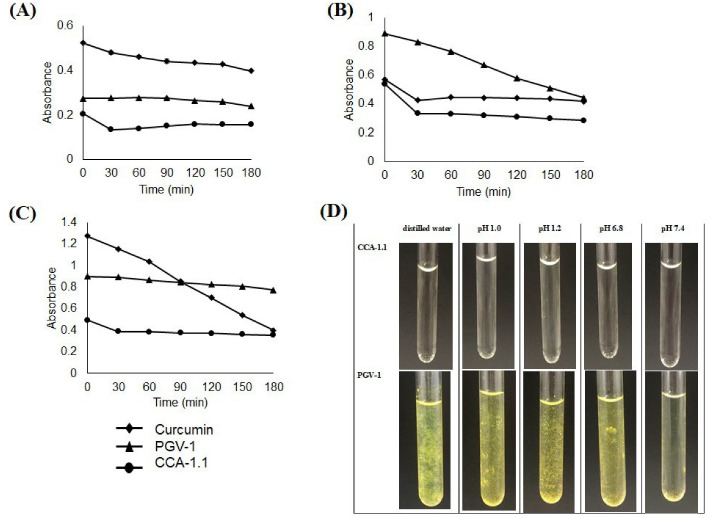
Stability test of curcumin, PGV-1, and CCA-1.1. The samples were dissolved in DMSO then diluted with buffer pH 1.0 (A), pH 7.4 (B), distilled water (C) to reach the concentration of 100 μM and subjected for absorbance measurement at the respected time. Solution appearance of CCA-1.1 and PGV-1 with final concentration of 100 μM were documented after 48 hours of storage at ambient temperature (D).

The evaluation of solubility and stability of CCA-1.1 were conducted in the buffer and non-buffer medium. We used buffer in pH 1.0 (which represents pH in the stomach), pH 7.4 (which is defined as pH situation in lumen also in blood and colon), and distilled water to describe the differences of proton environment. The result showed that CCA-1.1 was more soluble than PGV-1 and curcumin at a concentration of 100 µM (Figure 6). We then observed all compounds’ stability in storage times under a light environment monitored by a UV spectrophotometer. We found that curcumin and PGV-1 tended to decrease the absorbance at 3 hours, but not for CCA-1.1, especially in pH 1.0 (acidic), but not in pH 7.4. We also determined the stability of both PGV-1 and CCA-1.1 after being stored at room temperature for 48 hours. We realized that the CCA-1.1 solution still looked clear, but the PGV-1 solution appeared with some sediment or crystal (Figure 6D), showing that PGV-1 was more unstable than CCA-1.1.

### Cytotoxicity of CCA-1.1 compared with PGV-1

The cytotoxic effect of CCA-1.1 was investigated in various breast cancer cell lines (MCF-7/HER2, 4T1, MCF-7, HCC1954, T47D), human leukemic cells (K562), human colon carcinoma cells (Caco2 & WiDr), and immortalized fibroblast cells (NIH-3T3). Cytotoxic assay on several cancer cell lines showed that the IC_50_ values of both PGV-1 and CCA-1.1 were revealed to be less than 10 µM (Table 3). Interestingly, CCA-1.1 was found to be more cytotoxic than PGV-1 on 4T1 and K562 cells. Although both compounds exhibited superior cytotoxic activities against cancer cells, it was still less toxic on non-cancerous NIH-3T3 cells with a concentration up to 50 µM indicating that both compounds provide high selectivity.

**Table 3:** Cytotoxicity of CCA-1.1 in comparison with PGV-1 against many types of cancer and non-cancerous cell line

**No.**	**Cell Line**	**IC**_50_ ** value (µM)**	
		**CCA-1.1**	**PGV-1**
1.	MCF-7/HER2	5	7
2.	4T1	3	4^2^
3.	MCF-7	< 10	6^31^
4.	HCC1954	6	3
5.	K562	0.293	0.450^1^
6	T47D	7	8
7.	MCF-7/EV	8	8
8.	Caco2	5	12
9.	WiDr	5	> 10
10.	NIH-3T3	> 50	> 50

## Discussion

Curcumin is already known as a natural compound that shows cytotoxic activities against several types of cancer cells but unstable in aqueous solution resulting in low bioavailability.^12,18^ Curcumin is easily degraded through breaking in alpha-beta diene bonds to generate aldehyde (vanillin) compound.^10^ In summary, curcumin possesses limitations such as low water solubility, slight absorption in the gut, and less stable *in vivo* experiment.^19,20^ Numerous attempts have been carried out to overcome curcumin problems by synthesizing the modified curcumin, like its analogs, derivatives, and pro-drugs, but without sufficient results.^1^ Moreover, although curcumin analog PGV-1 has superior cytotoxic properties than curcumin, it can have less perfect stability. The present research developed a new derivative compound from PGV-1 by reducing the ketone group into a hydroxyl group while retaining alpha-beta diene to achieve better physical-chemical properties and cytotoxic effects than PGV-1 and curcumin since the diene part has contributed to cytotoxic effect.^21,22^

The synthesis of CCA-1.1 by reducing the ketone group on PGV-1 produced a good result with a yield of 10% under PLC separation. Analysis using FT-IR showed a dramatic decrease in the peak at around 1620 cm^-1^, which indicates a change in the ketone to a hydroxy group without a change in the diene bonds. This synthesis result, although still not perfect, can already show their repeatability so that this method can be applied for further synthesis. The results collected can also be sufficient for other experiments that are needed in anticancer *in vitro* studies. However, the reaction conditions must be optimized to increase the yield of the product. The method can be improved by optimizing reaction time, reaction techniques, and concentrations, including the separation technique.^15^ Theoretically, NaBH_4_ can reduce the ketone group up to > 90% with more than 40% efficiency. Therefore, the yield may increase and reach up to 40%.^14^ Furthermore, in solvent-free environments with a contribution of wet SiO_2_ (30%), the reduction using NaBH_4_ can be performed effectively.^15^ Ireson et al^23^ also achieved several metabolic reductions of curcumin for metabolism studies of curcumin by reducing the ketone groups in curcumin.

This new compound shows several advantages compared to PGV-1 in several aspects. CCA-1.1 has better solubility in water solvents in various pH compared with PGV-1. During observation at pH 1.0; 7,4; also in distilled water, CCA-1.1 was two times more soluble than PGV-1 at observations of up to 48 hours where at a concentration of 100 µM, PGV-1 dissolved showed the presence of insoluble aggregates while the CCA-1.1 solution was still apparent (Figure 6). The PGV-1 solution at a 50 µM concentration even appeared clear (data are not shown). This phenomenon is comparable with the solubility of curcumin at 40 µM.^10^ Further chemical stability test exhibited that CCA-1.1 performed unchanged absorbance up to 3 hours but not for PGV-1, indicating that CCA-1.1 was more stable in an aqueous solution in an acidic environment than PGV-1. However, both compounds’ stability is still better than that of curcumin, which decreases in absorbance in less than 60 minutes.^10,11^ This result indicates that CCA-1.1 significantly increases the solubility and stability of PGV-1, making it a promising anticancer drug with minimal side effects.

Previous molecular docking studies highlighted the contribution of the two benzenes or its substituent of PGV-1 on the formation of hydrophobic or hydrogen bonds. Still, none of them mentioned the role of its cyclic ketone.^1,24,25^ Furthermore, hydrogen bonds’ appearance on the structure between the two benzenes increased the affinity of many curcumin derivatives on several cancer marker proteins.^18^ In the present study, we synthesized compound CCA-1.1 by modifying the cyclic ketone into cyclic alcohol to improve the binding affinity of PGV-1. CCA-1.1 performed comparable and even better in protein target interaction than PGV-1 on several marker proteins in *NF-κB* signaling and ROS metabolic enzymes. The *NF-κB* signaling pathway controls the expression of numerous genes that regulate cell proliferation, stress responses, and apoptosis.^26^ Inhibiting *NF-κB* signaling has potential therapeutic applications in cancer therapy.^27^ The docking result provides a compelling reason to develop this new compound (CCA-1.1) as an anticancer agent. Moreover, its ability to interact with ROS metabolic enzymes reinforces its anticancer mechanisms’ rational basis because increased ROS in the cell is believed to be one of the selective targets of anticancer agents.^28^ Several research types realize that a significant increase in ROS in cells can cause cancer cell death and rarely found in normal cells.^29^ These binding affinities of CCA-1.1 against several ROS metabolic enzymes *in silico* should be interesting to be clarified in a laboratory experiment with several physiological implications.

These molecular interacting models of CCA-1.1 would be essential to contribute its cytotoxic activities against cancer cells with the related markers. Our data support the phenomenon that CCA-1.1 performs onefold to twofold better cytotoxic activities on several cancer cells than PGV-1, which agrees with previous findings.^ 1–3,30^ In this regard, the two synthesized compounds were evaluated *in vitro* against some samples of breast cancer cell lines (MCF-7/HER2, 4T1, MCF-7, HCC1954, and T47D), human leukemic cells (K562), human colon carcinoma cells (Caco2 and WiDr), and immortalized fibroblast cells (NIH-3T3) as the reference of healthy cells. The high selectivity of CCA-1.1 (and PGV-1) against all of the tested cancer cells is an essential point of this result, realizing that CCA-1.1 is a promising candidate of an ideal anticancer agent. The most potent cytotoxic activity of both compounds can be highlighted on K562, a leukemic cell line with CCA-1.1 exhibited twice stronger than PGV-1. This phenomenon should be noted as an important finding as consistent with the previous result for PGV-1 that should be explored further for more deep studies.

Last but not least, we may also look at the cytotoxic results against colon and breast cancer cells, especially against TNBC and non TNBC cancer cells. This result is not extraordinarily different but essential to consider the alternative or choices for some specific cancer types. As various cells had other characteristics for their cell signaling, CCA-1.1 needed to be elaborated more for its molecular mechanism towards cancer cells. Cancer cells with HER2+ feature found in more than 30% cancer cases.^31^ Human Epidermal Growth Factor Receptor-2 (HER2) is a notable tyrosine kinase receptor responsible for the progression, proliferation, and metastasis of cancer.^32,33^ Regarding these results, whether CCA-1.1 modulates the HER2 signaling to inhibit cell growth will be the exciting focus for further research. Besides, the strong cytotoxic effect of CCA-1.1 against triple negative breast cancer cells, though only 15%-20% of cases led to elevated mortality in patients due to lack of specific target to eradicate these cancers,^34,35^ also need to be explored accordingly. Although using a limited number of cells (10 types of cell lines), this cytotoxic evaluation is sufficient to represent the target of cancer types as in the PGV-1 test as a reference compound. Later, we also identified possible mechanism activity from CCA-1.1 in breast cancer and colorectal cancer cells, including cell cycle arrest at the mitotic phase, induced a high amount of intracellular ROS that led to cell senescence, as well as attenuated cancer cells migration through inhibition of MMP-9 activity.^36-40^ Additionally, bioinformatic analysis of CCA-1.1 revealed several possible target genes, including *TP53, MAPK1*, and *ERBB2* in colorectal cancer.^41^ The overall results showed that CCA-1.1 might replace PGV-1. It has been reported that PGV-1 and being selective *in vitro* also show minimal side effects *in vivo*; therefore, CCA-1.1 is also expected to be safe. Even more, we should consider the superior solubility and stability of CCA-1.1 in the aqueous solution that may be important for the development of dosage form for clinical application. The ADME prediction of CCA-1.1 that looked better than PGV-1 also needs to be proven in the real experiment, especially with a specific formulation. Therefore, we still have to challenge ourselves to prepare a formula that performs much more stable in aqueous solution for intravenous administration.

## Conclusion

The CCA-1.1 can be prepared by reducing PGV-1 using NaBH_4_. This new compound exhibited better solubility and stability in aqueous solution with better cytotoxic activity towards several cancer cells than PGV-1. CCA-1.1 can be developed as a potent anticancer agent with fewer adverse effects.

## Acknowledgments

The authors acknowledge the Indonesian Ministry of Research and Technology through World Class Research (WCR) Program year 2020/2021 for financially support the study. We also thank Enago (https://www.enago.com) for the English-language editing of this manuscript.

## Ethical Issues

Not applicable.

## Conflict of Interest

The authors declare that there is no competing interest in preparing and publishing the manuscript.

## Supplementary files


Supplementary file 1 contains Figures S1-S3 and Table S1.
