# Supplementary material for: Preparation and Cytotoxic Evaluation of PGV-1 Derivative, CCA-1.1, as a New Curcumin Analog with Improved-Physicochemical and Pharmacological Properties
Source: Adv Pharm Bull. 2021 Jul 4;12(3):603–12. doi: 10.34172/apb.2022.063 (PMC9348534; doi:10.34172/apb.2022.063)
Supplement: Supplementary file 1 — contains Figures S1-S3 and Table S1. [file apb-12-603-s001.pdf]

Supplementary file 1

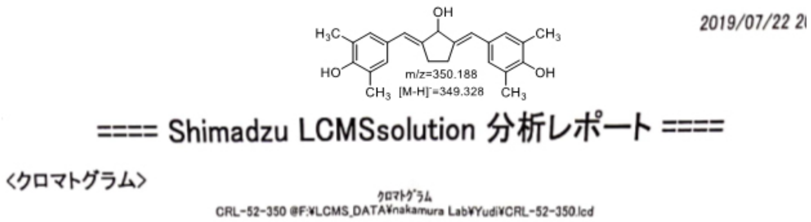

Figure S1. Mass spectrometric profile of CCA-1.1

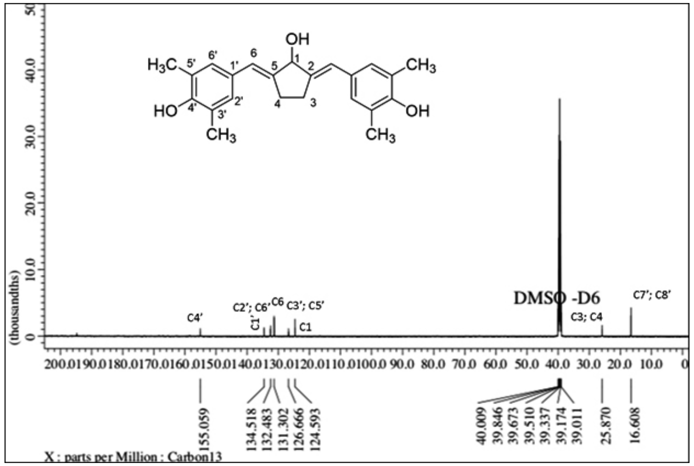

Figure S2. ^{13}C-NMR Spectrum of CCA-1.1

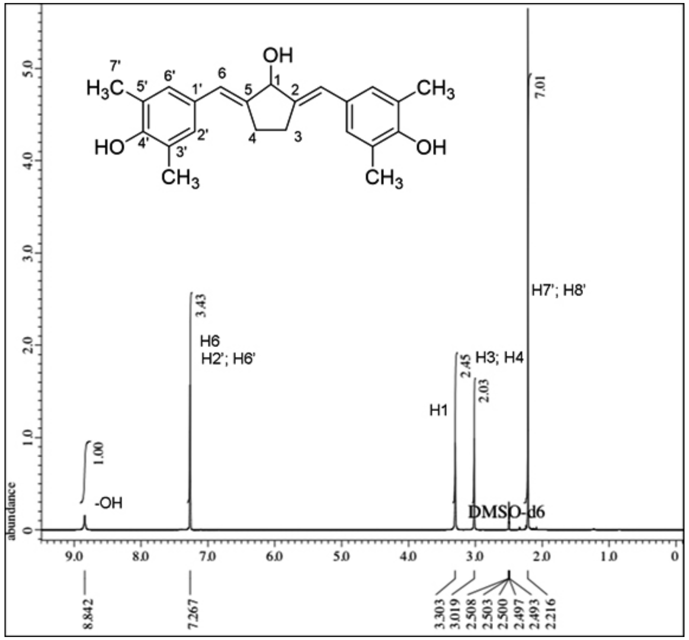

Figure S3. ^{1}H-NMR Spectrum of CCA-1.1

Table S1. RMSD value of the molecular docking study

Ligand	RMSD (Å)									
	HER2	EGFR	IKK	ERα	ERβ	NQO1	NQO2	AKR1C1	GST-P1	GLO1
PGV-1	0.721	1.288	1.539	1.241	1.539	1.728	1.003	1.641	1.156	1.964
CCA-1.1	1.862	1.189	1.854	1.778	1.165	1.727	1.261	1.288	1.089	0.821
